# Smith-Magenis Syndrome associated with Autism Spectrum Disorder with delayed diagnosis due to B12 deficiency: a case report

**DOI:** 10.1192/j.eurpsy.2024.921

**Published:** 2024-08-27

**Authors:** A. N. Orpay, H. A. Güler, S. Türkoğlu

**Affiliations:** Child and Adolescent Psychiatry, Selcuk University Faculty of Medicine, KONYA, Türkiye

## Abstract

**Introduction:**

Smith-Magenis syndrome (SMS) is a complex genetic disorder characterised by distinctive physical features, developmental delay, cognitive impairment and a typical behavioural phenotype. SMS is caused by interstitial 17p11.2 deletions (90%) involving multiple genes, including the retinoic acid-induced 1 gene (RAI1), or by pathogenic variants in RAI1 itself (10%).

**Objectives:**

In this case report, we present a case of Smith-Magenis syndrome with Autism Spectrum Disorder with karyotype 46,XX, 17p 11.2 gene deletion confirmed by Autism Spectrum Disorder, who was followed up in a paediatric neurology outpatient clinic with neuromotor developmental delay and whose diagnosis was delayed due to B12 deficiency. We also update scientific developments in Smith-Magenis syndrome.

**Methods:**

We describe an 18-month-old male with Smith-Magenis syndrome and Autism Spectrum Disorder who was seen in our paediatric psychiatric outpatient clinic and who received B12 replacement with developmental delay.

**Results:**

The patient was followed up in the paediatric neurology outpatient clinic with delay in neuromotor developmental milestones and this delay was thought to be due to B12 deficiency (B12<100 ng/L). The initial examination revealed delay in neuromotor and behavioural milestones, speech delay, wide and high nasal bridge and hypertelorism. Further physical examination revealed syndactyly of the second and third toes bilaterally and crossed lower teeth. Clinical and psychometric testing (Ankara Developmental Screening Inventory) by 2 consultants and 1 research assistant resulted in a diagnosis of intellectual disability and an additional diagnosis of Autism Spectrum Disorder due to social deficits that could not be explained by intellectual disability.

**Conclusions:**

Smith-Magenis syndrome is a well-known disorder involving the deletion of chromosome 17p11.2, which contains the RAI1 gene. This condition is associated with neuromotor and behavioural delay, as well as distinctive dysmorphic features. Clinicians should consider Smith-Magenis syndrome in the differential diagnosis for patients with delayed neuromotor and behavioural milestones, even in the presence of documented blood parameters (such as B12 deficiency) that may account for the delay.

**Disclosure of Interest:**

None Declared

